# Internalized homophobia and sexual risk behavior in men who have sex with men: The mediational role of sexual self-concept

**DOI:** 10.3389/fpsyg.2022.1007749

**Published:** 2022-10-14

**Authors:** Geraldy Sepúlveda-Páez, J. Francisco Santibañez-Palma, Rodrigo Ferrer-Urbina, Diego Atencio, Patricia Bucarei, Jenifer Castillo, Matías Fuentes, Bárbara Zumarán

**Affiliations:** Escuela de Psicología y Filosofía, Universidad de Tarapacá, Tarapacá, Chile

**Keywords:** internalized homophobia, sexual risk behavior, sexual self-concept, men who have sex with men, PHAT analysis

## Abstract

Men who have sex with men (MSM) are one of the populations most likely to be infected with human immunodeficiency virus (HIV) and acquired immunodeficiency syndrome (AIDS) worldwide. Sexual risk behaviors (SRB) are the main route of HIV transmission. Among the factors associated with SRB, internalized homophobia (rejection of one’s sexuality) is a risk factor unique for MSM. However, how this factor influences SRB is not clear. Therefore, the present study attempts to clarify the mechanism of action of the relationship between internalized homophobia on SRB based on the mediating effects of sexual self-concept. A study was conducted with 124 MSM living in Chile over 18 years of age (*M* = 24.4 and *SD* = 4.19). Through path analysis, it was observed that internalized homophobia has slight inverse effects on SRBs (multiple sexual partners and sexual activity under the influence of alcohol or drugs) when the sexual self-efficacy dimension acts as a mediating variable. These findings suggest that developing sexually transmitted infections (STIs) and HIV/AIDS prevention campaigns focused on MSM must highlight the development of a healthy sexual self-concept and address self-stigma.

## Introduction

Sexually Transmitted Infections (STIs), including Human Immunodeficiency Virus (HIV), have become a global public health challenge (Hemelaar et al., 2019; Mahy et al., 2021; Quinn, 2021), given the increase in the number of people infected with HIV (Joint United Nations Programme on HIV/AIDS [UNAIDS], 2021). HIV is prevalent primarily among young people aged 20–29, with a 3/1 male-to-female ratio (Institute of Public Health, 2016; Goldstein, 2019). Within high-risk groups, men who have sex with men (MSM) have the highest prevalence, at least 20 times higher than the general population (Stuardo, 2017; Diaz et al., 2019; Kabapy et al., 2020). The main way of HIV transmission is sexual risk behaviors (SRB) (Martinez et al., 2016; Luz et al., 2019; Coelho et al., 2021; Mulaudzi et al., 2022; Wei et al., 2022). Understood as sexual situations or practices that generate harm to one’s or others’ sexual health, for example, (1) sexual activity with multiple partners (Sönmez et al., 2021; Dong et al., 2022), (2) absence or misuse of condoms (Closson et al., 2018; Chu and Huang, 2020), (3) sexual activity under the influence of alcohol and drugs (Palfai and Luehring-Jones, 2021; Bustamante et al., 2022).

Different factors have been associated with SRB, including family values, peer group attitudes, alcohol and drug use, sex education, and social context (Jarrett et al., 2018; Valencia et al., 2018; Blondeel et al., 2021; Bozzini et al., 2021). Likewise, there is a large body of research focused on the individual’s psychological factors, which points to certain personality types and behaviors as a possible explanation for SRB (e.g., risk perception; sexual sensation seeking) (Danko et al., 2016; Xu et al., 2016; Gil-Llario et al., 2018; Wang et al., 2021).

Within these psychological factors, there is one that is specific to the lesbian, gay, bisexual, and transgender LGBT populations, and particularly to MSM, called internalized homophobia or internalized homonegativity (IH) (e.g., Berg et al., 2013; Parker et al., 2016; Rendina et al., 2017; Ramos et al., 2021; Michli and Jamil, 2022), both refers to a negative attitude toward one’s sexuality and is attributed to experiences of victimization and cultural heterosexism (Williamson, 2000), which, in extreme cases, can lead to rejection or shame of one’s sexual orientation, experiencing guilt, discrimination, having poor attachment to non-heterosexual others, and feeling dissatisfied with same-sex sexual activity (Meyer and Dean, 1998; Frost and Meyer, 2009; Cao et al., 2017; Meanley et al., 2020; Gill and Randhawa, 2021).

Some studies have shown a relationship between IH and SRB, noting that people who experience significant rejection of their sexuality tend to have more SRB, like inadequate condom use (Huebner et al., 2002; Morell-Mengual et al., 2017). However, it is unclear how internalized homophobia influences sexual risk behavior (Newcomb and Mustanski, 2011; Smolenski et al., 2011; Berg et al., 2015; Doyle and Molix, 2015; Puckett et al., 2017; Michael and Soskolne, 2020). Among the possible explanations found in the literature, it has been suggested that IH could affect the development of a healthy sexual self-concept (Hossain and Ferreira, 2019), propitiating risky sexual behaviors.

The self-concept refers to the dynamic and organized system of beliefs that an individual has about his or her identity, which is formed through experience and perceptions of the environment, playing an important role in multiples behaviors (Shavelson et al., 1976; Marsh and Shavelson, 1985; Jankowski et al., 2021). The literature often distinguishes between general self-concept and sexual self-concept; the latter is more specific and has been more frequently incorporated in sexuality research (Salehi et al., 2015a,b; Siu-ming et al., 2019). Sexual self-concept is defined as a multidimensional psychological construct that contains positive or negative cognitions and emotions about one’s sexuality (Deutsch et al., 2014; de Neve-Enthoven et al., 2022), which partially guides sexual behaviors (Sigre-Leirós et al., 2015; Potki et al., 2017; Emetu et al., 2020).

In the case of IH and its relationship with sexual self-concept, evidence suggests that those who are more prejudiced toward their sexuality tend not to express adequate identity integration (Rowen and Malcolm, 2003). Additionally, they exhibit a lower physical self-concept than heterosexual men (Shenkman and Toussia-Cohen, 2020) and also have a negative sexual self-image (Garcia et al., 2016; Puckett et al., 2018; Træen, 2018; Morell-Mengual et al., 2021; Foster et al., 2022). However, when there is adequate identity integration, levels of internalized homophobia decrease, as does depressive symptomatology (Li et al., 2021).

Although, studies attempting to explain the effects of IH on SRB are observed in the literature (e.g., Newcomb and Mustanski, 2011; Berg et al., 2015; Puckett et al., 2017; Michael and Soskolne, 2020). Explanations could be incomplete as the mechanism of action would not be direct, which makes it essential to develop new research exploring other strategies, as noted by Puckett et al. (2017).

The hypothesis in the following study proposes that self-stigma toward sexuality has a negative impact on sexual self-concept (Herek et al., 2015). This damage, in turn, would make people more prone to engage in risky sexual behaviors by decreasing the levels of sexual assertiveness to reject such behaviors (Ménard and Offman, 2009; Javier et al., 2018; Shafer et al., 2018; Van de Bongardt and de Graaf, 2020; Brasileiro et al., 2021). Therefore, this research aims to examine, through a mediation model, the mechanism of action of internalized homophobia on SRB, involving the indirect effects (i.e., mediation) of sexual self-concept in Chilean MSM.

## Materials and methods

A cross-sectional study with a descriptive correlational design was conducted (Ato et al., 2013). The sampling was non-probabilistic, mixing snowball and social network strategies (León and Montero, 2007).

### Participants

The sample consisted of 124 men who reported having sex with men in the last year. Only 124 cases were considered for the final sample out of 254 completed forms, given the inclusion criteria defined for the study (i.e., being male, having had sexual relations with other men in the last year, residing in Chilean territory, and having less than 10% of missing values).

Most respondents were residents of Arica (51.6%), followed by the city of Santiago de Chile (26.6%). The mean age was 24.4 years (*SD* = 4.19). A total of 65.3% of the participants reported not having a stable relationship, 26.6% had a partner, and 8.1% were living with a partner together. As for their educational level, 87.9% reported having completed or been in higher education, and 12.1% reported having completed secondary education. Over one-third (30.6%) reported being diagnosed with an STI, and 14 (11.3%) with HIV/AIDS. Regarding the sexual orientation of the participants, 66.1% reported being sexually attracted only to men, 23.4% reported being generally attracted to men. A further 7.3% reported being sometimes attracted to men and to a lesser extent 2.4% reported being generally attracted to women and finally only 0.8% reported being totally attracted to women in the sexual domain. On the other hand, 67.7% reported having sex only with men, 16.1% reported having sex usually with men, as well as 14.5% reported having sex sometimes with men.

#### Instruments

The Sexual Risk Behavior Scale (SRBS) (Ferrer-Urbina et al., 2019) is a 12-items self-report measure that assesses sexual behaviors at risk of STI or HIV/AIDS infection. Through four dimensions: sexual activity with multiple partners (items = 4); inappropriate or insufficient use of protective barriers (items = 4); (c) sexual activity under the influence of alcohol or drugs (items = 4); (d) knowledge of the partner’s sexual record (items = 4). The items are 4-level Likert attitudinal/behavioral statements (1 “Never”—4 “Always”). The version used has reported evidence of validity based on the internal structure of the test and adequate levels of reliability (ω > 0.8) (Ferrer-Urbina et al., 2019). The dimension of partners sexual history was excluded in a phase prior to data collection, as this dimension does not refer to risky sexual behavior itself. It has also been excluded from other published studies (Ferrer-Urbina et al., 2022).

Internalized Homophobia Scale (IHS) (Wagner, 1998) is a self-report measure composed of 20-items assessing the level of internalized homophobia This scale was conceived initially for homosexual men (Wagner, 1998); nonetheless, the present study gives it a more generalized use, involving a broader population such as MSM (i.e., who may be or identify as bisexual, pansexual, or even heterosexual regardless of their sexual behavior). The items are answered on a 5-point Likert scale (1 “Strongly disagree”—5 “Strongly agree”). The scale has reported adequate levels of reliability (α > 0.7) and evidence of validity, based on the internal structure of the construct, in Chilean samples (Pinto-Cortez et al., 2018).

The multidimensional scale of sexual self-concept (MSCS) (Ferrer-Urbina et al., 2019) is a 16-items self-report measure that assesses four dimensions whit 4-items each, which make up sexual self-concept: Sexual self-esteem, which refers to feelings about one’s sexuality (items = 4); sexual self-efficacy, concerning beliefs about one’s sexual ability (items = 4); assertive sexual behavior (items = 4); assertive sexual communication (items = 4), understood as the expressions of one’s sexual desires. The items are 4-level Likert behavioral/attitudinal statements (1 “Never”—4 “Always”; 1 “Strongly disagree”—4 “Strongly agree”). The scale has reported satisfactory levels of reliability in all its dimensions, and validity evidence, based on the internal structure of the test, through exploratory structural equation models in the Chilean sample (Ferrer-Urbina et al., 2022).

### Procedure

The Scientific Ethics Committee of the Universidad de Tarapacá approved the study within the framework of the FONDECYT Initiation Project No. 11170395.

The questionnaire was disseminated through social networks. An informed consent form was included at the beginning of the questionnaire explaining the study’s objectives, the voluntary nature of the application, and the confidentiality and anonymity of the data. Once participation was agreed upon, the participants answered three instruments (i.e., sexual risk behavior questionnaire, internalized homophobia questionnaire, and multidimensional sexual self-concept questionnaire) and a demographic section, with questions regarding age, and sexual orientation, among others. Once the questionnaire was completed, participants were invited to forward the form to their friends and close contacts to reach a significant number of people.

The response time for the questionnaire was approximately 15 min.

### Data analysis

The means and standard deviations of all dimensions were initially calculated. At the same time, internal consistency was examined using Cronbach’s alpha coefficient (α > 0.07). Additionally, univariate relationships were assessed using Pearson correlations, which were interpreted using Cohen’s effect size criteria (Cohen, 1988). Finally, a path analysis estimated indirect effects (i.e., mediation) was performed (Kline, 2016). Standardized regression coefficients (β) were assessed to determine the strength and direction of relationships between independent variables (i.e., internalized homophobia) and dependent variables (i.e., SRB in particular: multiple sexual partners; inadequate use of protective barriers; sexual intercourse under the influence of alcohol and drugs) while controlling for variables that comprise sexual self-concept (i.e., sexual self-esteem; sexual self-efficacy; assertive sexual behavior; assertive sexual communication). In addition, multiple regression assumptions were tested. Analyses were conducted using Jamovi software version 0.9.5.11 (The Jamovi Project, 2019).

## Results

The means and standard deviations of each of the dimensions, along with the reliability estimates, are presented in Table 1.

**TABLE 1:** Descriptive statistics for sample.

	Dimensions	M	SD	α
Independent variable	Internalized homophobia	1.94	0.584	0.858
Mediating variables	Sexual self-esteem	3.01	0.632	0.813
	Sexual self-efficacy	3.22	0.573	0.878
	Assertive sexual behavior	3.20	0.694	0.831
	Assertive sexual communication	3.20	0.609	0.809
Dependent variables	Multiplicity of sexual partners	1.71	0.545	0.782
	Inadequate use of protective barriers	2.22	0.502	0.610
	Sex under the influence of alcohol and drugs	1.61	0.543	0.780

M, mean; SD, Standard deviation; α = Cronbach’s alpha coefficient.

The scales are shown on a standard metric of 1–4, except for the IHS, which is on a scale of 1–5. Remarkably, the dimensions with the lowest means are sex under the influence of alcohol and drugs ($\bar{x}$ = 1.61), the multiplicity of sexual partners ($\bar{x}$ = 1.71), and internalized homophobia ($\bar{x}$ = 1.94), while all the dimensions of sexual self-concept present the highest means (> 3.00). Regarding the reliability estimates, most of the dimensions presented optimal or acceptable estimates, except for the variable: inadequate use of protective barriers which presented a lower value of 0.61.

In the case of the univariate effects of internalized homophobia on dimensions of sexual self-concept and sexual risk behavior, it is observed that internalized homophobia variable shows a moderate, inverse, and statistically significant effect on sexual self-efficacy (*r* = –0.368; *p* < 0.001), a small effect on assertive sexual communication (*r* = –0.198; *p* < 0.05) and sexual self-esteem (*r* = –0.266; *p* < 0.01). Also, a slight inverse effect (*r* = –0.246; *p* < 0.01) is observed in the inadequate use of protective barriers.

In the sexual self-concept and the dimensions that compose sexual risk behavior, a statistically significant, slight direct effect (*r* = 0.252; *p* < 0.01) is observed between sexual self-efficacy and the multiplicity of sexual partners, and a slight inverse effect (*r* = –0.207; *p* < 005) between assertive sexual behavior and sex under the influence of alcohol and drugs.

Concerning the evaluation of the assumptions for the development of a path model, it was found that there were no collinearity problems (tolerance > 0.4; VIF < 2) between independent and moderator variables. Moreover, the residuals of the dependent variables did not show severe deviations from normality (see Supplementary material). In addition, a posterior statistical power estimation was performed. The model used was one in which the direct effects between variables were equivalent to the largest effects observed in the correlation table (see Table 2); the power was 0.78 (Schoemann et al., 2017).

**TABLE 2:** Correlation coefficients between measures.

Variable	1.	2.	3.	4.	5.	6.	7.
Internalized homophobia	−						
Sexual self-esteem	−0.266**						
Sexual self-efficacy	−0.363***	0.570***					
Assertive sexual behavior	–0.149	0.188*	0.246**				
Assertive sexual communication	−0.198*	0.354***	0.363***	0.456***			
Multiplicity of sexual partners	–0.144	0.039	0.252**	–0.078	0.018		
Inadequate use of protective barriers	−0.246**	0.020	0.057	–0.022	0.030	0.252**	
Sex under the influence of alcohol and drugs	0.045	0.135	0.104	−0.207*	–0.094	0.328***	0.242**

***p < 0.001; **p < 0.01; *p < 0.05; α = Cronbach’s alpha coefficient.

Independent variable = internalized homophobia; mediating variables = sexual self-esteem; sexual self-efficacy; assertive sexual behavior and assertive sexual communication; dependent variables = multiplicity of sexual partners; Inadequate use of protective barriers; sex under the influence of alcohol and drugs.

A mediated pathway was developed to assess whether the effects of internalized homophobia on SRB are mediated by sexual self-concept. In this model, the mediating variables were sexual self-esteem, sexual self-efficacy, assertive sexual behavior, and assertive sexual communication. The results of the mediation analysis are shown in Table 3.

**TABLE 3:** Standardized and unstandardized estimates, of internalized homophobia on sexual risk behavior, mediated by sexual self-concept.

		Dependent variables models											
		
		Multiplicity of sexual couples				Inadequate use of protective barriers				Sex under the influence of alcohol and drugs			
				
		β_*std*_	SE	C.I. 95%		β_*std*_	SE	C.I. 95%		β_*std*_	SE	C.I. 95%	
										
				Lower	Upper			Lower	Upper			Lower	Upper
Internalized homophobia	Direct effect	–0.079	0.077	–0.235	0.069	−0.264**	0.082	–0.408	–0.07	0.084	0.122	–0.155	0.324
	Total	–0.144	0.083	–0.297	0.028	−0.245**	0.075	–0.358	–0.063	0.045	0.083	–0.122	0.206
Sexual self-steem	Indirect effect	0.038	0.033	–0.018	0.110	0.011	0.029	–0.048	0.072	–0.040	0.032	–0.108	0.022
Sexual self-efficacy	Indirect effect	−0.121**	0.042	–0.210	–0.039	0.003	0.034	–0.059	0.075	–0.047	0.033	0.015	0.015
Assertive sexual behavior	Indirect effect	0.020	0.019	–0.009	0.069	0.009	0.017	–0.018	0.049	0.032	0.022	0.083	0.083
Assertive sexual communication	Indirect effect	8.17e-4	0.022	0.036	0.054	–0.004	0.022	–0.058	0.036	0.015	0.022	0.064	0.064

β_*std*_, Betas standardized; SE, Standard; C.I, confidence intervals.

***p < 0.001; **p < 0.01; *p < 0.05.

It is observed that internalized homophobia has a direct, statistically significant, and inverse effect on the adequate use of protective barriers (β = –0.264, *p* < 0.01). Similarly, regarding indirect effects, it was observed that internalized homophobia had inverse effects on multiple sexual partners (β = –0.121, *p* < 0.01) when the sexual self-efficacy dimension acted as a mediating variable. No significant direct effects of internalized homophobia were found on the multiplicity of sexual partners or sex under the influence of alcohol or drugs. Nor were indirect effects of assertive sexual behavior, such as assertive sexual communication or sexual self-esteem, observed as mediating variables.

## Discussion

Employing a mediation model, this study aimed to examine the link between internalized homophobia and SRB through sexual self-concept in MSM. The results suggest that internalized homophobia has only slight inverse effects on the absence or misuse of condoms (i.e., a higher level of internalized homophobia is linked to a decrease in inappropriate use of protective barriers). However, when sexual self-efficacy acts as a mediating variable, an effect on the multiplicity of sexual couples is presented, which gives partial support to the study hypothesis since we only observed mediation in one of the 12 contrasted combinations (i.e., 3 dimensions of SRB × 4 dimensions of self-concept).

Regarding the effects of internalized homophobia on the dimensions that make up sexual self-concept (i.e., sexual self-esteem, sexual self-efficacy, assertive sexual behavior, assertive sexual communication), inverse effects were observed on sexual self-efficacy, sexual self-esteem, and assertive sexual communication. The higher the levels of internalized homophobia, the lower the perceived ability to achieve effective sexual behaviors and responses, communicate sexual preferences and needs, and have a more negative appraisal of one’s sexuality. This appraisal is consistent with available research, as previous studies have shown that those who are more prejudiced against their sexuality have negative self-concepts about their sexuality (Hossain and Ferreira, 2019).

However, in the case of internalized homophobia and SRB (i.e., SRB in particular: multiple sexual partners; inadequate use of the protective barriers; sexual relations under the influence of alcohol and drugs), inverse effects on the inadequate use of protective barriers are observed, which differs from what has been found in the literature, where it has been pointed out that higher levels of internalized homophobia would decrease control over one’s sexual behavior, making it more impulsive and risky (Puckett et al., 2017). However, in the sample studied, there seems to be an opposite effect since when the levels of rejection of one’s sexuality are higher, the proper use of condoms increases. A possible explanation for this finding could be that higher levels of internalized homophobia translate into higher levels of stigmatization and prejudice toward LGBT people, including beliefs such as that LGBT people are promiscuous and that homosexuality and transsexuality are directly related to HIV/AIDS (Barrientos et al., 2016).

In the mediational analysis, results shown that internalized homophobia has an indirect effect on SRB (i.e., multiple sexual partners) when it is mediated by sexual self-efficacy. This result is consistent with the findings of Shahar et al. (2020), who found no relationship between internalized homophobia and SRB in MSM, except when mediating variables such as self-efficacy and depression were considered. In this particular case, a possible explanation could be that people who have high levels of internalized homophobia tend to have a lower diversity of sexual partners, but only when they have low perceived self-efficacy, whereas, in those who have higher levels of sexual self-efficacy, internalized homophobia has no impact on partner diversity.

Although these findings show a minor role of IH on SRB, these results show that the direct effects of IH may be underestimated by not including other variables that may have relevant mediational effects. Additionally, the small effect sizes may be underestimated, given that the sample presented low mean levels of HI and SRB. The high mean levels of sexual self-concept, accompanied by low dispersion, may underrepresent the effects observed at more extreme values. Despite this, we consider that, our findings add to the emerging field of research on the mechanisms that influence and mediate internalized homophobia in sexual minorities such as MSM (Li et al., 2021; Munn and James, 2022), we emphasize the need to incorporate other mediational aspects to the study and understanding of HI (e.g., social support, age, gender) aspects that are not very frequent but that could contribute to a better understanding.

Finally, it is necessary to point out the limitations of population representativeness, both because of the small sample size and the non-probabilistic approach, so it is suggested that this study be replicated in other samples to increase the generalizability of these findings. Replication and clarification of these findings could be valuable for developing STI and HIV/AIDS prevention campaigns focused on MSM, highlighting the importance of promoting the development of a healthy sexual self-concept and combating stigma toward the MSM population.

## Conclusion

These results support the idea that internalized homophobia is not a relevant risk factor by itself. However, when associated with other aspects such as self-concept and self-perceived efficacy, it becomes detrimental for MSM, as in some cases it could generate different risk behaviors, specifically those addressed in this study referring to risky sexual behaviors (i.e., multiple partners). These results demonstrate the need to expand the field of research.

## Data availability statement

The raw data supporting the conclusions of this article will be made available by the authors, without undue reservation.

## Ethics statement

The studies involving human participants were reviewed and approved by the Comité de ética científico de la Universidad de Tarapacá. The patients/participants provided their written informed consent to participate in this study.

## Author contributions

RF-U contributed to conceptualization, review, and editing of methodology and was the main academic and research mentor. GS-P contributed to formal analysis, data curation, and writing the original draft. FS-P wrote the original draft and participated in the preparation of the original research concept. RF-U, GS-P, and FS-P participated in the review of the research proposal, prior to submission. DA, PB, JC, MF, and BZ worked to process the samples. All authors contributed to the article and approved the submitted version.

## References

[B1] Ato M. López-Garcia J. Benavente A. (2013). A classification system for research designs in psychology. *Ann. Psychol.* 29 1038–1059. 10.6018/analesps.29.3.178511

[B2] Barrientos J. Cárdenas M. Gómez F. Guzmán M. (2016). “Gay men and male-to-female transgender persons in chile: An exploratory quantitative study on stigma, discrimination, victimization, happiness and social well-being,” in *Sexual orientation and transgender issues in organizations*, ed. Köllen T. (Berlin: Springer), 253–270. 10.1007/978-3-319-29623-4_15

[B3] Berg R. C. Ross M. W. Weatherburn P. Schmidt A. J. (2013). Structural and environmental factors are associated with internalized homonegativity in men who have sex with men: Findings from the European MSM Internet Survey (EMIS) in 38 countries. *Soc. Sci. Med.* 78 61–69. 10.1016/j.socscimed.2012.11.033 23261257

[B4] Berg R. C. Weatherburn P. Ross M. W. Schmidt A. J. (2015). The relationship of internalized homonegativity to sexual health and well-being among men in 38 European countries who have sex with men. *J. Gay Lesbian Ment. Health* 19 285–302. 10.1080/19359705.2015.1024375 26692916 PMC4647863

[B5] Blondeel K. Dias S. Furegato M. Seuc A. Gama A. Fuertes R. (2021). Sexual behavior patterns and STI risk: Results of a cluster analysis among men who have sex with men in Portugal. *BMJ Open* 11:e033290. 10.1136/bmjopen-2019-033290 33483434 PMC7825267

[B6] Bozzini A. B. Bauer A. Maruyama J. Simões R. Matijasevich A. (2021). Factors associated with risk behaviors in adolescence: A systematic review. *Braz. J. Psychiatry* 43 210–221. 10.1590/1516-4446-2019-0835 32756805 PMC8023154

[B7] Brasileiro J. Widman L. Evans R. Javidi H. (2021). Social self-efficacy and sexual communication among adolescents in the United States: A cross-sectional study. *Sex. Health* 18 172–179. 10.1071/SH20221 33926613 PMC11926737

[B8] Bustamante M. J. Palfai T. P. Luehring-Jones P. Maisto S. A. Simons J. S. (2022). Cannabis use and sexual risk among MSM who drink: Understanding why more frequent cannabis users may engage in higher rates of condomless sex. *Drug Alcohol Depend.* 232:109282. 10.1016/j.drugalcdep.2022.109282 35066459 PMC8885905

[B9] Cao H. Zhou N. Fine M. Liang Y. Li J. Mills-Konce W. R. (2017). Sexual minority stress and the well-being of same-sex relationships: A meta-analysis of research prior to the national legalization of same-sex marriage in the U.S. *J. Marriage Fam.* 79 1258–1277. 10.1111/jomf.12415 28989184 PMC5627620

[B10] Chu J. H. Huang J. H. (2020). Psychosociobehavioral characteristics associated with high condomless anal intercourse intention: A comparison of receptive, versatile, and insertive MSM in Taiwan. *AIDS Care* 32 770–778. 10.1080/09540121.2019.1653433 31422670

[B11] Closson K. Dietrich J. J. Lachowsky N. J. Nkala B. Palmer A. Cui Z. (2018). Sexual self-efficacy and gender: A review of condom use and sexual negotiation among young men and women in sub-saharan Africa. *J. Sex Res.* 55 522–539. 10.1080/00224499.2017.1421607 29466024

[B12] Coelho L. E. Torres T. S. Veloso V. G. Grinsztejn B. Jalil E. M. Wilson E. C. (2021). The prevalence of HIV among men who have sex with men (MSM) and young MSM in Latin America and the Caribbean: A systematic review. *AIDS Behav.* 25 3223–3237. 10.1007/s10461-021-03180-33587242

[B13] Cohen J. (1988). *Statistical power analysis for the behavioral sciences*, 2nd Edn. Milton Park: Routledge. 10.4324/9780203771587

[B14] Danko M. Buzwell S. Earle M. (2016). Men at risk of HIV: Sexual sensation seeking, sexual compulsivity and sexual risk behavior among Australian MSM who frequently present for post-exposure prophylaxis. *Sex. Addict. Compulsivity* 23 324–341. 10.1080/10720162.2016.1140605

[B15] de Neve-Enthoven N. G. M. Callens N. Van Kuyk M. Verhaak C. M. Van der Ende J. Drop S. L. S. (2022). Sexual self-concept in women with disorders/differences of sex development. *Arch. Sex. Behav.* 51 2213–2229. 10.1007/s10508-021-02188-1 35362786 PMC9192466

[B16] Deutsch A. R. Hoffman L. Wilcox B. L. (2014). Sexual self-concept: Testing a hypothesized model for men and women. *J. Sex Res.* 51 932–945. 10.1080/00224499.2013.805315 23998689

[B17] Diaz Y. M. S. Orlando-Narvaez S. A. Crossbow-Arnal R. (2019). Risk behaviors toward HIV infection. A review of emerging trends. *Sci. Collect. Health* 24 1417–1426. 10.1590/1413-81232018244.02322017 31066843

[B18] Dong Y. Liu S. Xia D. Xu C. Yu X. Chen H. (2022). HIV infection risk prediction model among MSM in China: Validation and stability. *Int. J. Environ. Res. Public Health* 19:1010. 10.3390/ijerph19021010 35055826 PMC8776241

[B19] Doyle D. M. Molix L. (2015). Social stigma and the romantic relationship functioning of sexual minorities: A meta-analytic review. *Pers. Soc. Psychol. Bull.* 41 1363–1381. 10.1177/0146167215594592 26199218 PMC4575636

[B20] Emetu R. E. Brandt A. S. Forster M. (2020). Sexual self-concepts among sexual minority men with childhood sexual abuse histories. *J. Gay Lesbian Ment. Health* 25 294–316. 10.1080/19359705.2020.1838379

[B21] Ferrer-Urbina R. Leal-Soto F. Bravo N. Huaranca C. Perez J. Salinas T. (2019). Scale of risk behaviors, associated with STI/HIV-AIDS, for young Chileans. *Eur. Proc. Soc. Behav. Sci.* 60 800–809. 10.15405/epsbs.2019.04.02.99

[B22] Ferrer-Urbina R. Mena-Chamorro P. Halty M. Sepúlveda-Páez G. (2022). Psychological factors and sexual risk behaviors: A multidimensional model based on the chilean population. *Int. J. Environ. Res. Public Health* 19:9293. 10.3390/ijerph19159293 35954656 PMC9367853

[B23] Ferrer-Urbina R. Sepúlveda-Páez G. L. Henríquez D. T. Acevedo-Castillo D. I. Llewellyn-Alvarado D. A. (2019). Development and validity evidence of the multidimensional scale of sexual self-concept in a Spanish-speaking context. *Psicol. Reflex. Crit.* 32:22. 10.1186/s41155-019-0136-1 32027012 PMC6966990

[B24] Foster A. M. Rivas-Koehl M. M. Le T. H. Crane P. R. Weiser D. A. Talley A. E. (2022). Exploring sexual minority adults’ pathway to suicidal ideation: A moderated serial mediation model. *J. Gay Lesbian Ment. Health* 1–19. *v. 10.1080/19359705.2022.2036665

[B25] Frost D. M. Meyer I. H. (2009). Internalized homophobia and relationship quality among lesbians, gay men, and bisexuals. *J. Couns. Psychol.* 56 97–109. 10.1037/a0012844 20047016 PMC2678796

[B26] Garcia J. Parker C. Parker R. G. Wilson P. A. Philbin M. Hirsch J. S. (2016). Psychosocial implications of homophobia and HIV stigma in social support networks: Insights for high-impact HIV prevention among black men who have sex with men. *Health Educ. Behav.* 43 217–225. 10.1177/1090198115599398 27037286 PMC4973624

[B27] Gill S. Randhawa A. (2021). Internalised homophobia and mental health. *Indian J. Health Wellbeing* 12 501–504.

[B28] Gil-Llario M. D. Morell-Mengual V. Giménez-García C. Salmerón-Sánchez P. Ballester-Arnal R. (2018). Sexual sensation seeking: A validated scale for spanish gay. lesbian and bisexual people. *AIDS Behav.* 22 3525–3534. 10.1007/s10461-018-2182-6 29882049

[B29] Goldstein E. (2019). *Evolution of HIV/AIDS in chile and selected Latin American countries. Asesoría técnica parlamentaria, 13.* Available online at: https://www.bcn.cl/obtienearchivo?id=repositorio/10221/27105/2/BCN_VIHSIDA__en_Chile_y_America_Latina_EG_final.pdf (accessed May 20, 2022).

[B30] Hemelaar J. Elangovan R. Yun J. Dickson-Tetteh L. Fleminger I. Kirtley S. (2019). Global and regional molecular epidemiology of HIV-1, 1990-2015: A systematic review, global survey, and trend analysis. *Lancet Infect. Dis.* 19 143–155. 10.1016/S1473-3099(18)30647-9 30509777

[B31] Herek G. M. Gillis J. R. Cogan J. C. (2015). Internalized stigma among sexual minority adults: Insights from a social psychological perspective. *Stigma Health* 1 18–34. 10.1037/2376-6972.1.S.18

[B32] Hossain F. Ferreira N. (2019). Impact of social context on the self-concept of gay and lesbian youth: A systematic review. *Glob. Psychiatry* 2 51–78. 10.2478/gp-2019-0006

[B33] Huebner D. Davis M. Nemeroff C. Aiken L. (2002). The impact of internalized homophobia on HIV preventive interventions. *Am. J. Community Psychol.* 30 327–348. 10.1023/A:1015325303002 12054033

[B34] Institute of Public Health, (2016). Results confirmation of HIV infection in Chile, 2010 - 2015. *Bol. Vigilancia Lab.* 6. *p.

[B35] Jankowski T. Bak W. Miciuk U. (2021). Adaptive self-concept: Identifying the basic dimensions of self-beliefs. *Self Identity* 1–36. 10.1080/15298868.2021.1997796

[B36] Jarrett S. B. Udell W. Sutherland S. McFarland W. Scott M. Skyers N. (2018). Age of sexual initiation and sexual and health risk behaviors in Jamaican adolescents and young adults. *AIDS Behav.* 22(Suppl. 1), 57–64. 10.1007/s10461-018-2058-9 29476435 PMC6133291

[B37] Javier S. J. Abrams J. A. Moore M. P. Belgrave F. Z. (2018). Condom use efficacy and sexual communication skills among African American College women. *Health Promot. pract.* 19 287–294. 10.1177/1524839916676253 29451031

[B38] Joint United Nations Programme on Hiv/Aids [UNAIDS] (2021). *Global AIDS monitoring 2022.* Available at https://www.unaids.org/sites/default/files/media_asset/global-aids-monitoring_en.pdf (accessed May 20, 2022).

[B39] Kabapy A. F. Shatat H. Z. Abd El-Wahab E. W. (2020). Attributes of HIV infection over decades (1982-2018): A systematic review and meta-analysis. *Transbound. Emerg. Dis.* 67 2372–2388. 10.1111/tbed.13621 32396689

[B40] Kline R. B. (2016). *Principles and practice of structural equation modelling*, 4th Edn. New York, NY: The Guilford Press.

[B41] León O. G. Montero I. (2007). A guide for naming research studies in Psychology. *Int. J. Clin. Health Psychol.* 7 847–862.

[B42] Li F. Liao J. Sun X. Yang T. Li T. Wang Y. (2021). Does self-concept clarity relate to depressive symptoms in Chinese gay men? The mediating effects of sexual orientation concealment and gay community connectedness. *Sex. Res. Soc. Policy* 1–13. *v. 10.1007/s13178-021-00666-8

[B43] Luz P. M. Veloso V. G. Grinsztejn B. (2019). The HIV epidemic in Latin America: Achievements and challenges in treatment and prevention. *Curr. Opin. HIV AIDS* 14 366–373. 10.1097/COH.0000000000000564 31219888 PMC6688714

[B44] Mahy M. I. Sabin K. M. Feizzadeh A. Wanyeki I. (2021). Progress toward global HIV treatment targets and impact by 2020. *J. Int. AIDS Soc.* 24:e25779. 10.1002/jia2.25779 34546655 PMC8454678

[B45] Marsh H. W. Shavelson R. (1985). Self-concept: Its multifaceted and hierarchical structure. *Educ. Psychol.* 20 107–123. 10.1207/s15326985ep2003_1

[B46] Martinez O. Arreola S. Wu E. Muñoz-Laboy M. Levine E. C. Rutledge S. E. (2016). Syndemic factors associated with adult sexual HIV risk behaviors in a sample of Latino men who have sex with men in New York city. *Drug Alcohol Depend.* 166 258–262. 10.1016/j.drugalcdep.2016.06.033 27449272 PMC4983513

[B47] Meanley S. P. Stall R. D. Hawk M. E. Surkan P. J. Shoptaw S. J. Matthews D. D. (2020). Multifactorial discrimination, discrimination salience, and prevalent experiences of internalized homophobia in middle-aged and older MSM. *Aging Ment. Health* 24 1167–1174. 10.1080/13607863.2019.1594161 30938175 PMC7041891

[B48] Ménard A. D. Offman A. (2009). The interrelationships between sexual self-esteem, sexualassertiveness and sexual satisfaction. *Can. J. Hum. Sex.* 18 35–45.

[B49] Meyer I. H. Dean L. (1998). “Internalized homophobia, intimacy, and sexual behavior among gay and bisexual men,” in *Stigma and sexual orientation: Understanding prejudice against lesbians, gay men, and bisexuals*, ed. Herek G. M. (Thousand Oaks, CA: Sage Publications, Inc), 160–186. 10.4135/9781452243818.n8

[B50] Michael S. Soskolne V. (2020). Internalized homophobia and sexual risk behavior among HIV-infected men who have sex with men in Israel. *Soc. Work Health Care* 59 709–724. 10.1080/00981389.2020.1859045 33302820

[B51] Michli S. Jamil F. E. (2022). Internalized homonegativity and the challenges of having same-sex desires in the Lebanese context: A study examining risk and protective factors. *J. Homosex.* 69 75–100. 10.1080/00918369.2020.1809893 32910742

[B52] Morell-Mengual V. Gil-Llario M. D. Ballester-Arnal R. Salmerón-Sanchéz P. (2017). Spanish adaptation and validation of the short internalized homonegativity scale (SIHS). *J. Sex Marital Ther.* 43 298–305. 10.1080/0092623X.2016.1149128 27007255

[B53] Morell-Mengual V. Gil-Llario M. D. Fernádez-García O. Ballester-Arnal R. (2021). Factors associated with condom use in anal intercourse among Spanish men who have sex with men: Proposal for an Explanatory Model. *AIDS Behav.* 25 3836–3845. 10.1007/s10461-021-03282-0 33914210

[B54] Mulaudzi M. Kiguwa P. Zharima C. Otwombe K. Hlongwane K. Dietrich J. J. (2022). Sexual risk behaviours among young people in Soweto, South Africa, during national confinement by COVID-19. *Sex. Med.* 10:100487. 10.1016/j.esxm.2021.100487 35131540 PMC9023243

[B55] Munn M. James D. (2022). internalized homophobia and suicide ideation among sexual minority adults: The serial mediation of core self-evaluations and depression. *Arch. Sex Behav.* 31 1–14. 10.1007/s10508-022-02316-5 36044126

[B56] Newcomb M. E. Mustanski B. (2011). Moderators of the relationship between internalized homophobia and sexual risk behavior in men who have sex with men: A meta-analysis. *Arch. Sex. Behav.* 40 189–199. 10.1007/s10508-009-9573-8 19888643

[B57] Palfai T. P. Luehring-Jones P. (2021). How alcohol influences mechanisms of sexual risk behavior change: Contributions of alcohol challenge research to the development of HIV prevention interventions. *AIDS Behav.* 25 314–332. 10.1007/s10461-021-03346-1 34148189 PMC8616779

[B58] Parker R. D. Lohmus L. Mangine C. Rüütel K. (2016). Homonegativity and associated factors among men who have sex with men in Estonia. *J. Community Health* 41 717–723. 10.1007/s10900-015-0145-7 26728280

[B59] Pinto-Cortez C. Fuentes O. Quijada M. Salazar C. Guerra C. San Román R. (2018). Psychological distress as a mediator between internalized homophobia and suicidal risk in Chilean men. *Behav. Psychol. Psicol. Conductual* 26 529–546.

[B60] Potki R. Ziaei T. Faramarzi M. Moosazadeh M. Shahhosseini Z. (2017). Bio-psycho-social factors affecting sexual self-concept: A systematic review. *Electron. Physician* 9 5172–5178. 10.19082/5172 29038693 PMC5633209

[B61] Puckett J. A. Mereish E. H. Levitt H. M. Horne S. G. Hayes-Skelton S. A. (2018). Internalized heterosexism and psychological distress: The moderating effects of decentering. *Stigma Health* 3 9–15. 10.1037/sah0000065

[B62] Puckett J. A. Newcomb M. E. Garofalo R. Mustanski B. (2017). Examining the conditions under which internalized homophobia is associated with substance use and condomless sex in young msm: The moderating role of impulsivity. *Ann. Behav. Med.* 51 567–577. 10.1007/s12160-017-9878-0 28124182 PMC5526743

[B63] Quinn T. C. (2021). Forty years of AIDS: A retrospective and the way forward. *J. Clin. Invest.* 131:e154196. 10.1172/JCI154196 34523618 PMC8439598

[B64] Ramos S. R. Lardier D. T. Jr. Opara I. Turpin R. E. Boyd D. T. Gutierrez J. I. Jr. (2021). Intersectional effects of sexual orientation concealment, internalized homophobia, and gender expression on sexual identity and HIV risk among sexual minority men of color: A pathway analysis. *J. Assoc. Nurses AIDS Care* 32 495–511. 10.1097/JNC.0000000000000274 34101701 PMC8221709

[B65] Rendina H. J. Gamarel K. E. Pachankis J. E. Ventuneac A. Grov C. Parsons J. T. (2017). Extending the minority stress model to incorporate HIV-positive gay and bisexual men’s experiences: A longitudinal examination of mental health and sexual risk behavior. *Ann. Behav. Med.* 51 147–158. 10.1007/s12160-016-9822-8 27502073 PMC5299076

[B66] Rowen C. J. Malcolm J. P. (2003). Correlates of internalized homophobia and homosexual identity formation in a sample of gay men. *J. Homosex.* 43 77–92. 10.1300/J082v43n02_05 12739699

[B67] Salehi M. Azarbayejani A. Shafiei K. Ziaei T. Shayegh B. (2015a). Self-esteem, general and sexual self-concepts in blind people. *J. Res. Med. Sci.* 20 930–936. 26929756 10.4103/1735-1995.172764 PMC4746865

[B68] Salehi M. Kharaz Tavakol H. Shabani M. Ziaei T. (2015b). The relationship between self-esteem and sexual self-concept in people with physical-motor disabilities. *Iran. Red Crescent Med. J.* 17:e25359. 10.4103/1735-1995.172764 25763279 PMC4341498

[B69] Schoemann A. M. Boulton A. J. Short S. D. (2017). Determining power and sample size for simple and complex mediation models. *Soc. Psychol. Pers. Sci.* 8 379–386. 10.1177/194855061771506

[B70] Shafer A. Ortiz R. R. Thompson B. Huemmer J. (2018). The role of hypermasculinity, token resistance, rape myth, and assertive sexual consent communication among college men. *J. Adolesc. Health* 62 S44–S50. 10.1016/j.jadohealth.2017.10.015 29455717

[B71] Shavelson R. J. Hubner J. J. Stanton G. C. (1976). Self-concept: Validation of construct interpretations. *J. Educ. Res.* 46 407–441. 10.3102/00346543046003407

[B72] Shenkman G. Toussia-Cohen Y. (2020). Physical self-concept and its association with depressive symptoms among gay men and lesbian women and their heterosexual counterparts. *Sex Roles* 83 114–125. 10.1007/s11199-019-01092-2

[B73] Sigre-Leirós V. Carvalho J. Nobre P. (2015). Preliminary findings on men’s sexual self-schema and sexual offending: Differences between subtypes of offenders. *J. Sex Res.* 53 204–213. 10.1080/00224499.2015.1012289 26219334

[B74] Siu-ming T. Phyllis K. S. W. Cherry H. L. T. Kan Kwok D. Lau C. D. (2019). Sexual compulsivity, sexual self-concept, and cognitive outcomes of sexual behavior of young Chinese Hong Kong males with compulsive sexual behavior: Implications for intervention and prevention. *Child. Youth Serv. Rev.* 104:104400. 10.1016/j.childyouth.2019.104400

[B75] Smolenski D. J. Stigler M. H. Ross M. W. Rosser B. R. (2011). Direct and indirect associations between internalized homonegativity and high-risk sex. *Arch. Sex. Behav.* 40 785–792. 10.1007/s10508-010-9705-1 21221754 PMC3418675

[B76] Sönmez I. Folch C. Lorente N. Berg R. C. Thurlby N. Schmidt A. J. (2021). Influence of internalized homonegativity on sexual risk behaviors of men who have sex with men in Spain. *Sex. Cult.* 26 932–950. 10.1007/s12119-021-09925-7 PMC10903703 38596388

[B77] Stuardo V. (2017). Forgotten prevention: The reemergence of HIV in Chile. *Rev. Chilena Infectol.* 34 419–420. 10.4067/s0716-10182017000400419 29165529

[B78] The Jamovi Project (2019). *Jamovi. (Version 1.0) [Computer Software].* Available at https://www.jamovi.org (accessed June 15, 2022).

[B79] Træen B. (2018). Predictors of internalized homonegativity in Norwegian men who have sex with men (MSM). *Scand. Psychol.* 5 1–21. 10.15714/scandpsychol.5.e2

[B80] Valencia R. Wang L. Y. Dunville R. Sharma A. Sanchez T. Rosenberg E. (2018). Sexual risk behaviors in sexual minority male adolescents: A systematic review and meta-analysis. *J. Prim. Prev.* 39 619–645. 10.1007/s10935-018-0525-8 30446869 PMC6267112

[B81] Van de Bongardt D. de Graaf H. (2020). Youth’s socio-sexual competences with romantic and casual sexual partners. *J. Sex Res.* 57 1166–1179. 10.1080/00224499.2020.1743226 32338540

[B82] Wagner G. (1998). “Internalized homophobia scale,” in *Handbook of sexuality-related measures*, eds Terri A. Fisher D. Davis C. M. Yarber W. L. (London: Routledge), 399–400.

[B83] Wang W. Yan H. Duan Z. Yang H. Li X. Ding C. (2021). Relationship between sexual sensation seeking and condom use among young men who have sex with men in China: Testing a moderated mediation model. *AIDS Care* 33 914–919. 10.1080/09540121.2020.1808156 32811183

[B84] Wei L. Tian J. Guo M. Zhu B. Jiang Q. Yu B. (2022). Trajectories of sexual risk behaviors and associated factors among young men who have sex with men in China. *Front. Public Health* 10:854616. 10.3389/fpubh.2022.854616 35387185 PMC8978629

[B85] Williamson I. R. (2000). Internalized homophobia and health issues affecting lesbians and gay men. *Health Educ. Res.* 15 97–107. 10.1093/her/15.1.97 10788206

[B86] Xu W. Zheng L. Liu Y. Zheng Y. (2016). Sexual sensation seeking, sexual compulsivity, and high-risk sexual behaviours among gay/bisexual men in Southwest China. *AIDS Care* 28 1138–1144. 10.1080/09540121.2016.1153587 26924809

