# Supplementary material for: Internalized homophobia and sexual risk behavior in men who have sex with men: The mediational role of sexual self-concept
Source: Front Psychol. 2022 Oct 14;13:1007749. doi: 10.3389/fpsyg.2022.1007749 (PMC9614244; doi:10.3389/fpsyg.2022.1007749)
Supplement: Supplementary file 1 [file Table_1.DOCX]

| **Table 1.**  S**upplementary**  *Collinearity Statistics.* | | | | | |
| --- | --- | --- | --- | --- | --- |
|  |  |  |  |  |  |
| **Variable** | | **VIF** | | **Tolerance** | |
| 1. Multiplicity of sexual partners |  | 1.27 |  | 0.789 |  |
| 2. Inadequate use of protective barriers |  | 1.11 |  | 0.905 |  |
| 3. Sex under the influence of alcohol and drugs |  | 1.25 |  | 0.802 |  |
| 4. Sexual self-esteem |  | 1.61 |  | 0.623 |  |
| 5. Sexual self-efficacy |  | 1.71 |  | 0.583 |  |
| 6. Assertive sexual behavior |  | 1.34 |  | 0.749 |  |
| 7. Assertive sexual communication |  | 1.44 |  | 0.696 |  |
| ***Note:*** VIF = Variance Inflation Factor. |  |  |  |  |  |
|  | | | | | |

**Figure 1**
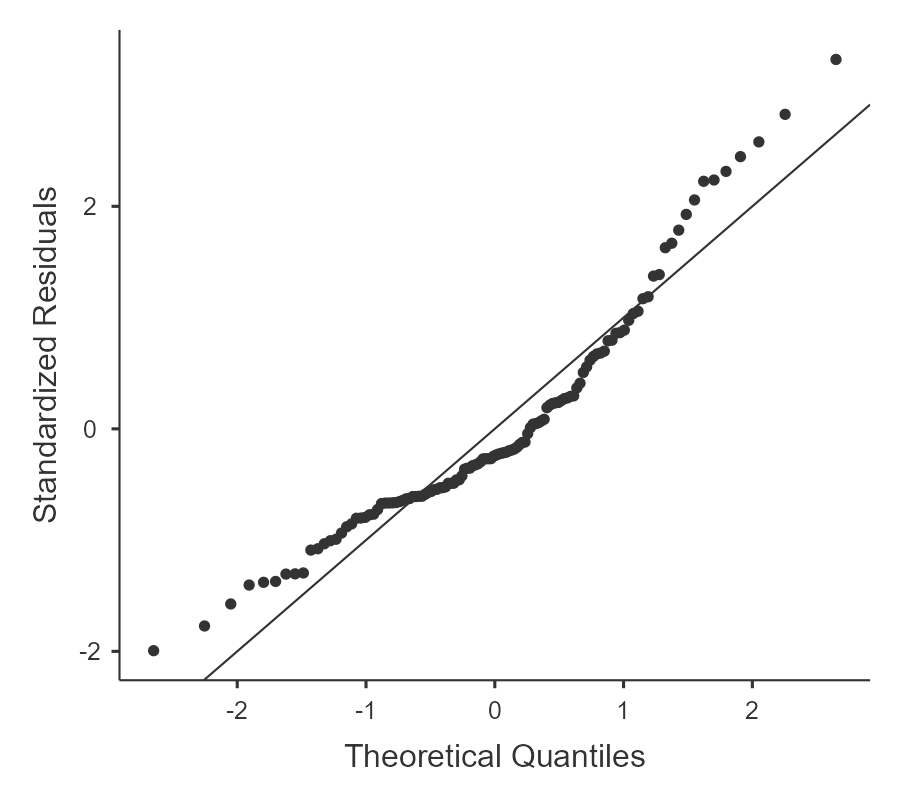
. Supplementary
